# Stereotactic ablative radiotherapy for comprehensive treatment of oligometastatic tumors (SABR-COMET): Study protocol for a randomized phase II trial

**DOI:** 10.1186/1471-2407-12-305

**Published:** 2012-07-23

**Authors:** David A Palma, Cornelis J A Haasbeek, George B Rodrigues, Max Dahele, Michael Lock, Brian Yaremko, Robert Olson, Mitchell Liu, Jason Panarotto, GwendolynHMJ Griffioen, Stewart Gaede, Ben Slotman, Suresh Senan

**Affiliations:** 1 Department of Radiation Oncology, London Regional Cancer Program, 790 Commissioners Rd. E, London, ON, Canada, N6A4L6; 2 Department of Radiation Oncology, VU University Medical Center, Amsterdam, Netherlands; 3 Department of Radiation Oncology, British Columbia Cancer Agency, Vancouver, Canada; 4 Department of Radiation Oncology, Ottawa Regional Cancer Centre, Ottawa, Canada

**Keywords:** Oligometastases, Stereotactic radiotherapy, Quality of life, Cancer, Survival

## Abstract

**Background:**

Stereotactic ablative radiotherapy (SABR) has emerged as a new treatment option for patients with oligometastatic disease. SABR delivers precise, high-dose, hypofractionated radiotherapy, and achieves excellent rates of local control. Survival outcomes for patients with oligometastatic disease treated with SABR appear promising, but conclusions are limited by patient selection, and the lack of adequate controls in most studies. The goal of this multicenter randomized phase II trial is to assess the impact of a comprehensive oligometastatic SABR treatment program on overall survival and quality of life in patients with up to 5 metastatic cancer lesions, compared to patients who receive standard of care treatment alone.

**Methods:**

After stratification by the number of metastases (1-3 vs. 4-5), patients will be randomized between Arm 1: current standard of care treatment, and Arm 2: standard of care treatment + SABR to all sites of known disease. Patients will be randomized in a 1:2 ratio to Arm 1:Arm 2, respectively. For patients receiving SABR, radiotherapy dose and fractionation depends on the site of metastasis and the proximity to critical normal structures. This study aims to accrue a total of 99 patients within four years. The primary endpoint is overall survival, and secondary endpoints include quality of life, toxicity, progression-free survival, lesion control rate, and number of cycles of further chemotherapy/systemic therapy.

**Discussion:**

This study will provide an assessment of the impact of SABR on clinical outcomes and quality of life, to determine if long-term survival can be achieved for selected patients with oligometastatic disease, and will inform the design of a possible phase III study.

**Trial registration:**

Clinicaltrials.gov identifier: NCT01446744

## Background

The oligometastatic disease state was first defined in 1995 and refers to an stage of disease where cancer has spread beyond the site of origin, but is not yet widely metastatic [1]. In such a state of limited metastatic disease burden, it is hypothesized that eradication of all sites of metastatic disease could result in long-term survival, or even cure, in a subgroup of patients [2]. Ablation of metastatic deposits can be achieved surgically, or through stereotactic ablative radiotherapy (SABR), a new radiotherapy technology that delivers very large, hypofractionated doses of radiotherapy with high precision to small tumor targets, with high rates of local control.

Clinical evidence to support the presence of an oligometastatic state is emerging in both the surgical and SABR literature, for several tumor types. In a study of over 5200 patients with lung metastases who underwent surgical resection, a 5-year survival of 36% was reported in patients who achieved a complete resection, much higher than would be expected for many patients with disseminated stage IV disease [3]. Similarly, in patients treated with SABR for 1-3 lung metastases from a variety of primary tumors, local control with SABR was 96% at 2-years, and 2-year survival was 39% [4]. Long-term survival has been demonstrated in patients treated for oligometastases with surgery or SABR at several other tumor sites, including liver, brain, bone, and adrenal metastases [2,5-8]. However, even after such treatment, the risk of further metastases after ablative treatment is high, up to 60-80% in some studies [4,5]. In some cases, SABR can be used for further salvage at newly progressive sites [9].

Despite the apparent achievement of long-term survival with ablative treatment for oligometastatic disease, the level of evidence to support such treatments is weak in many cases, often based on single-arm studies without appropriate controls [10]. Patients included in such reports are highly selected, based on good performance status and slow pace of tumor progression. It has been suggested that the long-term survival achieved with treatment of oligometastases is a result of the selection of fit patients with very slow-growing tumors, rather than the result of treatment intervention [11].

Randomized trials are therefore necessary to establish the utility of ablative treatment of oligometastatic disease [10,12], but such randomized trials are rare. One such completed randomized trial, Radiation Therapy and Oncology Group Trial 9508, compared whole brain radiotherapy (WBRT) with WBRT + stereotactic treatment for patients with 1-3 brain metastases, and found an overall survival advantage only in patients with a single metastasis and those patients in the most favorable baseline recursive partitioning analysis (RPA) prognostic group [13]. Patients with inferior baseline prognostic factors did not achieve a survival benefit from stereotactic treatment. At least one other randomized trial investigating the oligometastatic paradigm has recently opened: in 2010, the Pulmonary Metastasectomy in Colorectal Cancer (PulMiCC) trial was launched, comparing metastatectomy with best supportive care in patients with pulmonary metastases from colorectal cancer [14].

It is unclear if all patients with oligometastatic disease benefit from SABR, in terms of improved local control, improved survival or improved quality of life. Although SABR generally results in ablation of each metastatic target, patients remain at high risk of further metastatic progression. Results from SABR for treatment of oligometastases in published studies appears promising, but these promising results may be due to patient selection, rather than treatment intervention, and are based on comparisons with historical controls. The benefit of comprehensive treatment of oligometastases can only be demonstrated conclusively in the context of a randomized trial.

## Methods/design

**Figure 1:**
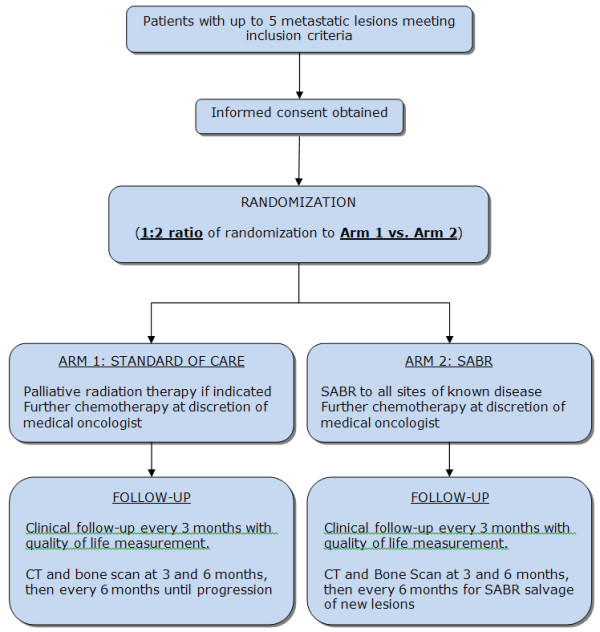
** Study design.** Patients with be randomized in a 1:2 ratio between Arm 1 (Standard of care) vs. Arm 2 (SABR).

This study is designed as a randomized phase II study. Patients will be randomized between current standard of care treatment (Arm 1) vs. standard of care treatment + SABR (Arm 2) to sites of known disease (Figure 1). Patients will be randomized in a 1:2 ratio to Arm 1 vs. Arm 2, respectively. This study has been approved by the Ontario Cancer Research Ethics Board (#11-030), in compliance with the Helsinki Declaration.

The randomized phase II design is required for 3 reasons: First, the randomization will provide an appropriate control group to serve as a comparator for the experimental arm. Historical or contemporaneous non-randomized controls would not be appropriate due to the multitude of biases that could be introduced by patient selection and other confounders. Second, a small sample size will allow for adequate power to assess for an early overall survival difference, quality of life, and to evaluate toxicity in the SABR arm. Third, the results will allow for a decision as to whether a multi-institutional phase III trial is warranted, and inform the design of such a trial.

### Objectives

To assess the impact of a comprehensive oligometastatic SABR treatment program on overall survival and quality of life in patients with up to 5 metastatic cancer lesions, compared to patients who receive standard of care treatment alone.

#### Primary endpoint

· Overall Survival

· Defined as time from randomization to death from any cause

#### Secondary endpoints

· Quality of life

· Assessed with the Functional Assessment of Cancer Therapy: General (FACT-G)

· Toxicity

· Assessed by the National Cancer Institute Common Toxicity Criteria (NCI-CTC) version 4 for each organ treated (e.g. liver, lung, bone)]

· Progression-free survival

· Time from randomization to disease progression at any site or death

· Lesional control rate

· Number of cycles of further chemotherapy/systemic therapy

### Inclusion criteria

· Age 18 or older

· Willing to provide informed consent

· Histologically confirmed malignancy with metastatic disease detected on imaging. Biopsy of metastasis is preferred, but not required.

· ECOG performance status 0-1

· Controlled primary tumor

· defined as: at least 3 months since original tumor treated definitively, with no progression at primary site

· All sites of disease can be safely treated based on criteria below

· Maximum 3 metastases in any single organ system (i.e. lung, liver, brain, bone), and the total number of metastases must be 5 or less. For example, a patient with two liver metastases and two lung metastases is eligible.

· Life expectancy >6 months

· Not a candidate for surgical resection at all sites: surgery to all sites not recommended by multidisciplinary team, or unfit or declining surgery

· Prior chemotherapy allowed but no chemotherapy (cytotoxic or molecularly targeted agents) therapy 4 weeks prior to first fraction of radiotherapy, during radiotherapy, or for two weeks after last fraction. Hormonal therapy is allowed.

· Patients with metastases that have been previously treated (e.g. prior resection, RFA or radiotherapy):

· If that previously treated metastasis is controlled on imaging, the patient is eligible for this study and that site does not need treatment

· If that previously treated metastasis is NOT controlled on imaging:

· If the previous treatment was surgery, the patient is eligible if that site can be treated by SABR

· If the previous treatment was radiotherapy or RFA, the patient is ineligibile.

· Patient presented at multidisciplinary tumor board or radiotherapy departmental quality-assurance rounds, with consensus opinion that entry into the study is appropriate.

### Exclusion criteria

· Serious medical comorbidities precluding radiotherapy

· Bone metastasis in a femoral bone

· Patients with 1-3 brain metastasis and no disease elsewhere (these patients should not be randomized but treated with stereotactic radiotherapy as per results of randomized trials)

· Prior radiotherapy to a site requiring treatment

· Complete response to first-line chemotherapy (i.e. no measurable target for SABR)

· Malignant pleural effusion

· Inability to treat all sites of active disease

· Clinical or radiologic evidence of spinal cord compression OR tumor within 3 mm of spinal cord on MRI.

· Dominant brain metastasis requiring surgical decompression

· Pregnant or lactating women

### Evaluation and randomization

Prior to randomization, a complete history and physical examination by the treating radiation oncologist is required. Histologically confirmation of malignancy is required, with metastatic disease detected on imaging. Biopsy of metastasis is preferred, but not required.

Patients must be restaged within 12 weeks prior to randomization, including brain CT or MRI imaging (for tumor sites with propensity for brain metastasis); and CT neck/chest/abdomen/pelvis with bone scan. PET-CT is only required for specific evidence-based indications, as defined by the Ontario Health Insurance Program, and in such cases the CT neck/chest/abdomen/pelvis and bone scan are not required. For other indications, at the discretion of the treating oncologists, PET-CT scans may be done but are not required. MRI spine is required for patients with vertebral or paraspinal metastases.

Patients with liver metastases are also required to have adequate liver function tests (AST, ALT, GGT, alkaline phosphatase). A negative pregnancy test is required for women of child-bearing age.

The study will employ a 1:2 randomization between Arm 1:Arm 2 (Figure 1). The sample size allows for one stratification factor at randomization: number of metastatic sites (1-3 vs. 4-5). Randomization will occur in permuted blocks of nine.

## Interventions

### Arm 1

Radiotherapy for patients in the standard arm should follow the principles of palliative radiotherapy as per the individual institution, with the goal of alleviating symptoms or preventing imminent complications. Patients in this arm should not receive stereotactic doses or radiotherapy boosts.

Treatment recommendations for patients in Arm 1 are as follows: For brain metastases, whole brain radiotherapy (i.e. 20 Gy in 5 fractions, 30 Gy in 10 fractions); for lung metastases, palliative radiotherapy as per 2011 consensus guidelines (i.e. 8 Gy in 1 fraction, 20 Gy in 5 fractions, 30 Gy in 10 fractions)[15]; for bone metastases, palliative radiotherapy as per 2011 consensus guidelines (i.e. 8 Gy in 1 fraction (most common), 20 Gy in 5 fractions, 30 Gy in 10 fractions)[16]; for liver metastases, 20 Gy in 5 fractions if standard institutional practice.

For Arm 1, Treatment planning is to be done using CT simulation or conventional simulation (fluoroscopy) as per individual institutional practice. Simple beam arrangements, such as parallel opposed beams, are favored wherever possible.

### Arm 2

For Arm 2, all treatments in this study are based on current protocols in clinical use at the LRCP and VUmc for treatment of lung [17], liver [18], brain [19,20], and spinal column [21] metastases. The guiding principle for radiotherapy is to achieve disease control but to minimize any potential adverse impact on quality of life. Concurrent chemotherapy or targeted therapy at the time of radiotherapy is not permitted within the 4 weeks prior to SABR. Hormone therapy is permitted.

Doses and fractionations by tumor site are shown in Table 1. Treatment will be setup using reproducible positioning, verified using an on-line protocol, for all patients in this study. Immobilization may include a custom immobilization device, such as thermoplastic shell or Vac-lok^TM^ bag, as per individual institutional practice when delivering SABR. Some centers do not use immobilization devices and have demonstrated high degrees of accuracy; this is acceptable in this study.

**Table 1:** Dose and fractionations by site

**Tumor Location**	**Description**	**Total Dose (Gy)**	**Number of fractions**	**Dose per fraction (Gy)**	**Frequency**
**Lung**	Tumors 3 cm or less surrounded by lung parenchyma	54	3	18	Every second day
	Abutting chest wall or >3 cm	55	5	11	Every second day
	Within 2 cm of mediastinum or brachial plexus	60	8*	7.5	Every second day
**Bone**	Any bone except femur	35 Gy	5	7	Daily
	Vertebral body: additional options	16-20 Gy *OR*	1	16-20	Single dose
		30 Gy	3	10	Every second day
**Brain Metastases**	If whole brain treated, then simultaneous boost to each lesion	40 Gy to metastases 20 Gy whole brain (optional)	5	8 Gy to lesion 4 Gy WBRT	Daily
**Liver**	LRCP site: Dose is based on calculated normal tissue probability of <5%				Every second day
	Other sites	45-60	3-8	7.5-15	Every second day
**Adrenal**		60 Gy	8	7.5	Every second day

*If esophageal dose constraints cannot be met, 12 fractions should be used.

All patients in Arm 2 will undergo planning CT simulation. 4-dimensional CT will be used for tumors in the lungs or liver. Axial CT images will be obtained throughout the region of interest. For all lesions, the gross tumor volume (GTV) will be defined as the visible tumor on CT and/or MRI imaging +/- PET. No additional margin will be added for microscopic spread of disease, consistent with current protocols (i.e. Clinical Target Volume [CTV] = GTV). For vertebral lesions, the entire vertebral body may be considered the CTV, as per institutional practice. A Planning Target Volume (PTV) margin of 2-5 mm will be added depending on site of disease, immobilization, and institutional set-up accuracy: 2 mm margins may be used for spinal stereotactic treatments, 2 mm for brain tumors, and 5 mm for other sites. Organs at risk visible in the planning CT scan will be contoured. Constraints for 1-, 3- and 5-fraction regimens are taken from Timmerman *et al.* 2008 [22], whereas equivalent doses were calculated for other fractionation schemes.

For spinal lesions, a pre-treatment MRI is required to assess the extent of disease and position of the cord. This must be fused with the planning CT scan. A Planning Organ at Risk Volume (PRV) expansion of 2 mm will be added to the spinal cord, and dose constraints for the spinal cord apply to this PRV.

It is strongly recommended that dose constraints not be exceeded. If a dose constraint cannot be achieved due to overlap of the target with an organ at risk, the fractionation can be increased or the target coverage compromised in order to meet the constraint. It is strongly recommended that in cases where the target coverage is compromised in order to meet the constraint, the mean dose delivered to the GTV should be at least 80% of the nominal dose in Table 1. All such cases of dose reduction or target coverage compromise must be approved by the local PI prior to treatment. For vertebral tumors, an adequate PRV of 2 mm must be added to the spinal cord, and the dose constraints apply to this PRV.

For lung tumors, doses are prescribed to approximately the 80% isodose line surrounding the PTV, resulting in a hotspot of 120-140%; the latter should fall within the GTV. 95% of the PTV should be covered by the prescription dose, and 99% of the PTV should be covered by 90% of the prescription dose.

For other tumor sites, doses are prescribed to approximately the 100% isodose level and 95% of the PTV should receive 95% of the prescription dose. Doses will be corrected for tissue inhomogeneity. Several non-overlapping 6/10 MV beams (on the order of 7-11 beams) or 1-2 VMAT arcs combined possibly with a few non-coplanar beams is recommended. Non-coplanar beams can be used to reduce 50% isodose volume for un-gated treatments. Coplanar beams are recommended for respiratory-gated treatment.

For lung or liver metastases, each metastasis can be treated with a separate isocenter if metastases are well-separated. Since most metastases are treated every other day (Table 1), when two metastases are treated, these can be done on alternating days to reduce the daily time required on the linear accelerator (e.g. Monday/Wed/Friday for one target, and Tues/Thurs/Mon for another). For brain metastases, all the metastases should be treated at the same time. For bone metastases, if multiple metastases can be imaged and localized at the same time, they can be treated at the same time, otherwise, they can be treated on alternate days.

The sequencing of tumor sites is at the discretion of individual physicians, but in general should begin with the brain, due to risks associated with progression, followed thereafter by liver, lung, and bone.

### Quality assurance (Arm 2)

In order to ensure patient safety and effective treatment delivery, a robust quality assurance protocol is incorporated. The following requirements must be completed for each patient:

· Prior to treatment, each patient must be discussed at quality assurance (QA) rounds.

· All radiotherapy plans must meet target dose levels for organs at risk (Tables 2). Prior to plan approval, the dose to each organ at risk must be verified by the physicist or treating physician. It is strongly recommended that dose constraints not be exceeded.

· All dose delivery for intensity-modulated plans (including arc-based treatments) will be confirmed before treatment by physics staff.

· Cone-beam CT (CBCT) will be used to verify patient positioning immediately prior to treatment. Ideally, direct tumour localization should be performed for stereotactic treatments of soft tissues. For gated SABR treatments, direct tumour localization will be performed by matching the tumour position with the ROI defined by IGTV_CBCT. This will be followed by a gated 2D-kV in the AP plane to verify the gating window. In the absence of direct tumour localization, reliable soft tissue surrogates are recommended. A repeat CBCT will be done 25 minutes after the first, if delivery requires more than 25 minutes. A final CBCT may be done after completion of treatment.

**Table 2:** Follow-up schedule

	**Before Treatment**	**Years 3-5**		**Years 3-5**
		**Every 3 months**	**Month 3, 6, 12, 18 and 24**	**Every 6 months**
History and Physical	X	X		X
Baseline staging investigations (see text)	X			
CT head, chest, abdomen, pelvis			X	X
Bone Scan			X	X
Toxicity Scoring		X		X
FACT-G QOL scoring	X	X		X

### Quality assurance for centres joining study

Prior to opening the study, each participating research centre will be required to send to one of the Principal Investigators a mock treatment plan for the anatomic sites that will be treated (e.g. lung, brain, liver, adrenal), to ensure that the treatment plans are designed in compliance with the protocol. The principal investigators will provide pertinent CT datasets. Each participating research centre can choose which tumor sites will be treated at their individual centre (i.e. some centres may only choose to treat a subset of the eligible metastatic sites).

### Chemotherapy

Patients treated with prior chemotherapy are eligible for this study, however, no chemotherapy agents (cytotoxic, or molecularly targeted agents) are allowed within the period of time commencing 4 weeks prior to radiotherapy (conventional or SABR) lasting until 2 weeks after the last fraction. Hormonal therapy is allowed**.** Use of chemotherapy schemes containing potent enhancers of radiation damage (e.g. gemcitabine, adriamycin) are discouraged within the first month after SABR.

### Further radiotherapy for progressive disease at new metastatic sites

Patients in Arm 1 who develop new, untreated metastatic deposits can receive palliative radiotherapy for any new such sites of progression. Patients in Arm 2 who develop new, untreated metastatic deposits should be considered for SABR at those sites, if such deposits can be treated safely with SABR, and if the treating institution offers SABR for that body site. If SABR is not possible, then palliative RT can be delivered if indicated.

### Follow-up

Patients will be seen every three months post-randomization for the first two years, and every six months until 5 years after treatment (Table 2). At each visit, a history and physical examination will be conducted by the oncologist, and CTC-AE toxicities recorded. The FACT-G Quality of life questionnaire is to be completed at each visit.

CT head, chest, abdomen and pelvis, and bone scans will be repeated at 3 and 6 months, then every six months. Patients randomized to Arm 2 will be considered SABR for salvage if new sites of disease develop. Additional Imaging or laboratory investigations should be carried out at the discretion of the oncologist, based on findings in the history or physical, and additional treatment (e.g. further chemotherapy) is at the discretion of the oncologists.

### Measurement of response

Overall survival will be measured as time until death from any cause, and progression-free survival as time to either progression or death, whichever occurs first. Lesion response will be evaluated in this study using the international criteria proposed by the Response Evaluation Criteria in Solid Tumors (RECIST) Committee (http://ctep.info.nih.gov/guidelines/recist.html). The sum of the longest diameter (LD) for all target lesions will be calculated and reported as the baseline sum LD. The baseline sum LD will be used as reference by which to characterize the objective tumor response.

## Statistical analysis

### Sample size

This study will employ a randomized phase II design, to conduct a preliminary and non-definitive randomized comparison between the control and experimental arms. The study will aim to detect a signal in improved overall survival that would be used to design a phase III study to definitely compare survival outcomes between the two groups. The study will therefore be designed with alpha = 0.20 and beta = 0.20 (as recommended for phase II randomized studies [23]). It is estimated that the median survival of the control group after randomization in this study will be 9 months.

There will be a 1:2 randomization between Arm 1 and Arm 2. In order to detect a 6-month improvement in median survival from 9 months to 15 months with SABR, a total of 93 patients (31 in Arm 1 and 62 in Arm 2) will be needed. Assuming a 5% rate of loss to follow-up, a total of 99 patients will be accrued (33 in Arm 1 and 66 in Arm 2). The study projects accrual over 48 months with 12 months of additional follow-up. Accrual targets are as follows: 20 patients in year 1, and 25-30 patients in years 2, 3, and 4.

### Data analysis

Patients will be analyzed in the groups to which they are assigned (intention-to-treat).

#### Primary endpoint

Survival will be calculated using the Kaplan-Meier method with differences compared using the stratified log-rank test. Pre-planned subgroup analysis will occur based on stratification variables. A Cox multivariable regression analysis will be used to determine baseline factors predictive of survival and allow for assessment of time to failure data important in this patient group.

#### Secondary endpoints

Quality of life at 6 months will be measured using FACT-G scores, with differences between groups tested using the Student’s *t*-test. Differences in rates of grade 2 or higher toxicity between groups will be tested using the Fisher’s Exact Test. Differences in progression free survival will be tested using the stratified log-rank test. Differences in the number of cycles of further chemotherapy/systemic therapy will be tested using the Student’s *t*-test.

### Data safety monitoring committee

The DSMC will meet annually after study initiation and after 50 patients are accrued to review toxicity outcomes. If any grade 3-5 toxicity is reported, the DSMC will review the case notes to determine if such toxicity is related to treatment. If the DSMC deems that toxicity rates are excessive (>25% grade 3 toxicity, or >5% grade 4 or 5 toxicity), then the DSMC can, at its discretion, recommend cessation of the trial, dose adjustment, or exclusion of certain treatment sites that are deemed as high-risk for complications.

The DSMC will conduct one interim analysis once 50 patients are accrued. For this analysis, the DSMC will be blinded to the identity of each treatment arm, but OS data will be presented for each arm. The DSMC will recommend stopping the trial if there is an OS difference that is statistically significant with a threshold of p < 0.001 using the log-rank test. Furthermore, if the median OS among all patients is substantially different than estimated in the sample size calculation, the DSMC can recommend increasing or decreasing the target accrual in order to maintain statistical power.

## Discussion

Although the aggressive treatment of patients with oligometastatic disease has become more common over the last decade [24], considerable equipoise still exists as to whether such aggressive treatments improve overall survival, or whether the long-term survival seen in single-arm studies is due to patient selection and treatment of slow-growing, favorable tumors [25]. Although SABR and surgical resection of oligometastases are generally safe, there is a risk of toxicity, and a small risk of treatment-related mortality [26,27]. Since patients with metastatic disease have traditionally been considered to be best served by palliative treatments, it is incumbent upon physicians to demonstrate that the escalation of treatment (with attendant risks of side effects or complications) is associated with gains in survival and/or quality of life.

This multicenter, international study aims to accrue 99 patients and aims to provide preliminary evidence to assess the impact of a comprehensive oligometastatic SABR treatment program on overall survival and quality of life. Data from this study may be used to inform the design of a phase III study, and will lead to a better understanding of the oligometastatic paradigm.

## Abbreviations

SABR, Stereotactic ablative radiotherapy; WBRT, Whole brain radiotherapy; RPA, Recursive partitioning analysis; PulMiCC, Pulmonary metastasectomy in colorectal cancer; FACT-G, Functional assessment of cancer therapy: general; NCI-CTC-AE, National Cancer Institute Common Terminology Criteria for Adverse Events; CT, Computed tomography; PET, Positron emission tomography; MRI, Magnetic resonance imaging; AST, Aspartate transaminase; ALT, Alanine transaminase; GGT, Gamma glutamyl transpeptidase; LRCP, London regional cancer program; VUmc, Vrije University medical center; GTV, Gross tumor volume; CTV, Clinical target volume; PTV, Planning target volume; PRV, Planning organ at risk volume; PI, Principal investigator; VMAT, Volumetric modulated arc therapy; QA, Quality assurance; CBCT, Cone Beam CT; ROI, Region of interest; IGTV, Internal GTV; RECIST, Response evaluation criteria in solid tumors; LD, Longest diameter; DSMC, Data safety monitoring committee; OS, Overall survival.

## Competing interests

The authors declare that they have no competing interests.

## Author’s contributions

Study conception: DAP, CJAH, GBR, MD, ML, SS. Initial study design: DAP, CJAH, GBR, MD, ML, BY, RO, JP, BS, SS. Revision of study design and protocol: All authors Drafting and approval of final protocol and manuscript.

## Pre-publication history

The pre-publication history for this paper can be accessed here:

http://www.biomedcentral.com/1471-2407/12/305/prepub
